# Reclaiming the new era in global health: A proposal for an equity-centered paradigm

**DOI:** 10.1371/journal.pgph.0007379

**Published:** 2026-10-01

**Authors:** Pooja Yerramilli, Mickey Chopra

**Affiliations:** 1 Department of Medicine, New York University Grossman School of Medicine, New York, New York, United States of America; 2 Health, Nutrition and Population Global Practice, World Bank, Washington, District of Columbia, United States of America; New Zealand Institute of Public Health, UNITED KINGDOM OF GREAT BRITAIN AND NORTHERN IRELAND

## Abstract

Global health is facing unprecedented upheaval, with significant donor disruptions requiring a reconfiguration of the global health landscape. While recently proposed frameworks for the next phase of global health represent meaningful progress in grappling with this context, their central weakness is not technical but normative. In this Essay, we argue that if the new global health architecture is defined without meaningful interrogation of its central purpose, then the system will reproduce the very problems it aims to solve. On the other hand, explicitly centering health equity as the organizing principle of the new “global health” brings to the foreground the shared struggles and movements of marginalized communities worldwide. In doing so, it demands a more fundamental, global reckoning with the structural determinants – politics and power - that produce ill health across and within countries. From this logic flows substantive shifts in global health functions, predicated on a social-ecological model: from substitution to mutual aid; from technical assistance to knowledge sharing; from global public goods to multisectoral common goods for all; and from mechanisms that facilitate global coordination to ones that also critically address the distribution of power and resources.

## Introduction

In light of major funding disruptions, many experts have been reconceptualizing the next iteration of global health. Several prominent frameworks have proposed revised contexts and functions for a new global health system, while broader consultative processes have converged on principles of decentralization, new financing models, and expanded scope [1–3].

While analytically useful, these frameworks proceed without making their normative foundations explicit - primarily because they seek to adapt the pre-existing global health landscape to its current financial context. In doing so, they risk privileging questions of system design - how functions should be organized and delivered - over more fundamental questions concerning the purpose of global health. This risks reproducing a technocratic logic in which questions of how power and resources are distributed are displaced by questions of efficiency and coordination. Assuming consensus on ends such as continuity and sustainability presents existing power and resource distributions as given, shifting attention away from whether those distributions are just, historically produced, or politically contestable.

Even at its most fundamental level, the classification of countries illustrates this distortion: by sorting countries into donors and recipients, frameworks reliant on such categories naturalize the very inequities they claim to address. The geographic distribution of need does not map neatly to traditional country categories, such as income or stability. Hookworm in rural Alabama, leprosy in northern Australia, and tuberculosis in London are not “L/MIC problems that appeared in high-income countries (HIC)” - they are manifestations of the same structural determinants of marginalization, disinvestment, and inadequate infrastructure that produce poor health outcomes globally [4–6]. Frameworks reliant on traditional country classifications risk rendering marginalized populations worldwide invisible.

We argue that a more explicit departure is required: a questioning of the assumptions underlying the current global health architecture, a refocusing of its central purpose as health equity across and within countries, and a treatment of equity as the organizing principle of the new global health architecture. As such, building off of existing literature and evidence on health equity, its social and structural determinants, and decolonization, we propose an approach to defining global health contexts and functions, which shifts from “thin” multilateralism - coordination on shared goods where state interests converge, as in pandemic surveillance - to “thick” multilateralism - redistributive equity frameworks where interests structurally diverge [Fig 1]. Therefore, unlike previous efforts to center equity in health – whether the Alma-Ata Declaration (1978) or the Sustainable Development Goals (2015) – our proposed framework moves beyond “community participation in health [without addressing] questions around power and control” to collective action for health by centering these very questions [7].

**Fig 1:**
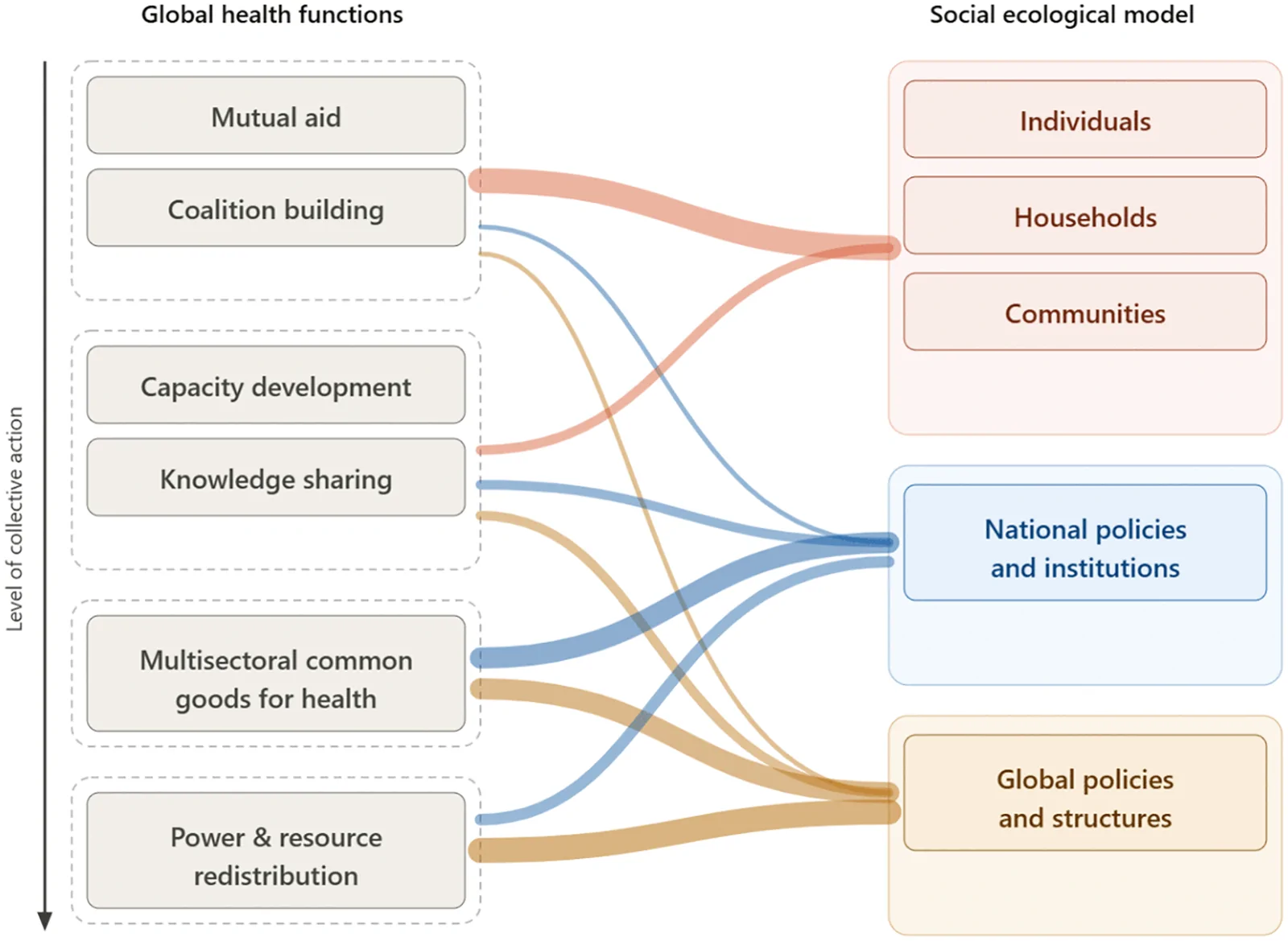
Functions based on a social ecological model for global health. Note: The four main functions of the new proposed architecture map to three interacting levels of the social ecological model (SEM). All SEM levels (even those not visualized, like subnational) influence all functions; however, each line represents which SEM levels most impact each function, and therefore where the target of action or reform should focus, with the line thickness corresponding to the strength of the relationship.

## The fundamental problem with traditional “country categories”

Prominent proposals for the new global health system center around the nation-state as the organizing unit and hence default to country categories to define the nature of engagement. Yet the categories utilized are historically contingent and obscure the heterogeneity across and within countries within the same classification. Specifically, these frameworks organize the global health system around categories based on income (i.e., GDP) and stability that: (a) are products of a global economic and political order whose assumptions are not interrogated; (b) obscure the very inequities they claim to address; and (c) predetermine which functions are appropriate for which contexts based on one-dimensional definitions. Therefore, such categories are simplistic and ill-equipped to meet today’s most pressing global challenges.

### Country income classifications

Fundamentally, organizing around country-income categories risks naturalizing inequity across countries as a starting condition rather than an outcome to be challenged. The World Bank’s country-income classifications were initially defined in 1989 based on Gross National Income (GNI) per capita, as operational thresholds [8] – but have since become widely adopted into broader global health and development vernacular to define both problems and solutions. Low and middle-income countries (L/MICs) “are” low- and middle-income; we are not prompted to ask why or how this came to be, or whether global health’s purpose should include challenging these classifications. This neglects historical critiques of the enduring global economic and political systems which produce health inequities. Nkrumah’s structural critiques of post-independence economic dependency illuminate why country categories map so closely onto colonial-era development hierarchies: the economic structures that produce “low-income” status were shaped by colonial extraction designed to benefit the metropole [9]. Fanon’s writings on colonial medicine established that health systems in colonized territories were constructed to serve extraction rather than population health - a structural condition that current income-classification schemes were not designed to correct [10].

This complacency is further apparent in the passive acceptance of GNI or GDP per capita as an objective measurement of countries’ “development” status. In 2013, redefining R&D expenditures as a fixed asset rather than an intermediate input produced overnight increases in US GDP, revealing that GDP itself reflects normative and political choices [11]. The per-capita component further skews reality. Botswana, which has a low population density, becomes an upper-middle-income country, obscuring some of the “lower-middle income country” (LMIC)-type problems it faces due to its diamond-dependence and resultant economic and commercial vulnerabilities; vast and diverse geography, making many communities “last mile communities;” and significant internal inequities [12]. India’s LMIC classification obscures both extreme poverty in some states and substantial capacity in others.

The consequences of such simplified groupings are clear in the data: between 2000–2020, between-country income inequalities decreased, but within-country income inequalities nearly doubled, with a significant amount of variation within each country-income or geographic category [13].

### Stability classifications

Proposed global health frameworks further treat humanitarian settings as exceptional rather than as predictable outcomes of structural conditions, foreclosing questions about what drives given contexts to become “humanitarian” or “fragile” in the first place [1,2]. By avoiding engagement with upstream “structural determinants” of conflict, such frameworks assume that if a geography is classified as a humanitarian setting, substitutive services should and can be delivered. Yet recent experience suggest that this assumption is often untenable: the delivery of humanitarian assistance is itself shaped - and at times constrained or obstructed - by power and political dynamics producing conflict to begin with [14].

Moreover, definitions of humanitarian crises are imprecise, leaving significant discretion in determining which communities qualify for substitutive services [15,16]. They often lend themselves to a false stable/humanitarian binary, with many countries one climate shock or political crisis away from reclassification. They do not account for “stable countries” which have humanitarian emergencies and crises emerge within them. For example, while the United States as a whole has remained classified as “stable,” Hurricane Katrina prompted over 130 countries to offer humanitarian support to the country; more recently, Mexico sent firefighters and emergency personnel to respond to the California wildfires in 2025 [17,18]. People in need of humanitarian assistance in such “stable” countries often remain uncounted: by the end of July 2026, over 300,000 people have been internally displaced by the wildfires in France and Spain, but these individuals, like those impacted by extreme weather events like the Australian bushfires of 2019–2020, are not captured in any global humanitarian dashboards or reports.

### Implications of existing country categories

These nuances suggest that country categories are not merely descriptive but performative - they structure which questions can be asked and what solutions appear logical. They obscure the mechanisms - state capacity, within-country distribution, historical legacies - that mediate the relationship between income and health outcomes, erasing the vast differences between countries within the same category [19,20]. In doing so, reliance on traditional country categories perpetuates “North-South” directionality of knowledge (and aid), reflecting, rather than correcting, the colonial ordering of who produces knowledge and who receives it [21].

Looking beyond the traditional nation-state model shows that the same structural forces cause similar challenges for marginalized groups everywhere, whether in low, middle, or high-income countries. The same commercial determinants of health (CDOH) operate in beverage and food markets worldwide, requiring similar regulatory capacity-building [22]. The same patterns of racialized exclusion produce neglected tropical diseases in both Alabama and Sub-Saharan Africa, requiring similar WASH infrastructure investment [4]. The same dynamics of state abandonment occur in humanitarian contexts, “stable” L/MICs facing austerity, and HICs defunding public services for marginalized groups. When none of these similarities are visible in a framework organized by traditional country categories, global health is likely to perpetuate existing global power asymmetries.

## A new “equity centered” global health paradigm

### The normative case for health equity

Only an equity-centered global health paradigm can reflect and address the nuanced dynamics that define our collective health. Indeed, though global health has been framed around overlapping yet distinct agendas - from health security to economic growth to human rights - decades of evidence and literature, magnified by the COVID-19 pandemic, have demonstrated that improving health equity across and within boundaries – through action on social determinants including universal health coverage - is necessary to advance each of these agendas [23–25]

Yet, proposals for the “new global health architecture” *mention* within-country inequities, but fall short of explicitly defining them as symptoms of structural problems [1–3]. In these frameworks, this manifests as a reliance on traditional country categories to define eligibility for global health support, while rendering marginalized populations as implementation challenges that ought to be considered. This treatment of health equity merely as a cross-cutting issue, rather than the organizing principle of the global health system, risks reproducing the inequities that recent decades of global health investment have failed to resolve. A new global health system centered around health equity does the opposite: it builds the scaffolding required to uplift marginalized populations through collective movements that rebalance power across and within countries.

An equity-centered paradigm does not simply add marginalized populations to existing categories - it changes the unit of analysis. Where income-based classification asks “which country needs assistance?”, an equity lens asks “which populations, within any country, face structural barriers to health?” This shift has immediate practical consequences. It makes visible the high-burden populations in high-income countries currently excluded from global health programming. It recognizes the technical and knowledge assets within low-income countries currently discounted by a donor-recipient logic. It replaces stability as a binary condition with a spectrum of structural risk - one shaped by political economy, climate exposure, and historical dispossession rather than by whether a country currently hosts a declared emergency.

Using the Rasanathan et al (2025) framework as a starting point, we show how an equity-centered approach might meaningfully grapple with power asymmetries and lead to a revised set of global health functions [Fig 1]. Rather than country contexts, this revised framework is organized around a social ecological model, identifying how loci of power interact across global, national, and community structures to impact the individual. This approach can inform who needs what, regardless of where they are located, and empower all individuals to take responsibility for our collective health. Instead of grading the level of cooperation needed for each function, this revised framework emphasizes the collective action required to shift power, including to strengthen the existing technical functions of global health [1].

In the proposed paradigm, shown in Fig 1, four main functions map to three interacting levels of the social ecological model (SEM). Each line represents which SEM levels most influence each function, and therefore where the target of action or reform should be, with the line thickness corresponding to the strength of the relationship. While global and national policies and institutions can support “mutual aid and coalition building,” most of this function occurs at the individual, household, and community level. “Capacity development and knowledge sharing” is conceptualized as a function facilitated by structures across all levels. Both “multisectoral common goods for health” and “power and resource redistribution” require high-level governance and coordination structures primarily at national and global levels. Each progressive function requires increasing levels of collective action to facilitate; because mutual aid largely operates outside existing systems, this function can be enabled with relatively lower levels of collective action to overcome structural opposition than the large-scale reforms or disruptions needed to enable power and resource redistribution.

### From substitution to coalition building and mutual aid

Across all contexts, there are circumstances in which the state disavows or is unable to fulfill its responsibilities to its population. This requires collective action to hold responsible entities accountable and, in the interim, mutual aid that operates outside or in parallel with structures that have failed - centering mutual responsibility and empowerment rather than unidirectional aid.

These contexts may be traditional “humanitarian crises,” but they may also extend beyond this definition to other contexts where traditional institutions fail to provide services to the people – for example communities excluded from or marginalized from health systems. The former may include conflicts in which collective action is required to hold warring entities to account, and in which mutual aid, including but not limited to traditional humanitarian response mechanisms, may be the most direct and impactful way of getting resources on the ground. The latter may include mutual aid to support families facing catastrophic health expenditures (e.g., crowdfunding platforms) or communities targeted by the state (e.g., undocumented immigrants), while advocating for more inclusive policies, including universal health coverage.

### From technical assistance to capacity development and knowledge sharing

The shift from technical assistance to capacity development and knowledge sharing recognizes that knowledge does not flow in a single direction. Recent disruptions to public health institutions in several high-income countries demonstrate that health system fragility is not exclusive to L/MICs. Knowledge sharing, underpinned by enabling structures, should run in all directions: community-oriented primary care models developed in L/MICs offer insights for HICs [26]; L/MIC expertise with public health emergencies can inform HIC preparedness [27]; and civil society-driven public health reforms - such as Mexico’s sugar-sweetened beverage tax - offer replicable models of collective action worldwide [28].

### From Global Public Goods (GPGs) to multisectoral common goods for health

Current frameworks acknowledge GPGs but narrowly scope them in ways that neglect broader common goods for health, which can include both GPGs and common-pool resources (CPR). CPR - rivalrous but with broad social externalities, such as systems and programs that deliver health services - make the normative case for global health to support health systems strengthening. More importantly, GPG and CPR frameworks grapple with power differently – while GPGs invoke supranational, top-down coordination and exertion of authority, CPR often center community or local levels and bottom-up authority [29]. Safeguarding common goods for health require both global coordination and grassroots solutions, including for core technical functions like disease surveillance or emergency response mechanisms worldwide.

Multisectoral common goods extend this logic to non-health agendas – such as climate change, conflict, and food security - that profoundly shape health [23]. This approach can build coalitions across sectors, rather than “single issue” or fragmented coalitions, to secure both health-specific common goods and the broader reforms necessary for equity.

### From transnational GPGs to accountability for power and resource distribution

The traditional framing of transnational health GPGs - focused on disease surveillance, control, and legal instruments - is insufficient to address structural inequities. An equity-focused vision demands explicit focus on how power and resources are distributed globally. This section provides a list of illustrative, not exhaustive, policies and reforms which can enable such power and resource redistribution.

Reforms of global health and development institutions are a prerequisite to any framework intent on delivering on equity. Such reforms begin with democratization of the World Health Organization, international financial institutions (IFI), and adjacent bodies, but extend to a critical appraisal of the global financing architecture and funding mechanisms writ large [30,31]. IFI conditionalities, such as IMF fiscal consolidation conditions, have historically required wage bill caps limiting health worker hiring and deficit reduction targets crowding out social expenditures [32]. Across L/MICs, debt service payments exceed public expenditures on education, health, and infrastructure combined [33]. Tackling this architecture head-on is central to funding the global health functions above.

Meaningful reform thus requires global financial and trade frameworks oriented toward health equity. In the face of unprecedented concentration of wealth among few individuals and corporations worldwide, the reform agenda centers progressive financing mechanisms, including international and national taxation of concentrated wealth, commensurate with the scale of global inequalities and global health needs [34]. Moreover, it demands global trade policies that subordinate commercial interests to public health imperatives, including robust regulation of private entities whose business models are structurally incompatible with human health and well-being – managing CDOH from intellectual property regimes that place essential medicines out of reach to industries producing tobacco, alcohol, and ultra-processed foods [22].

### Foundations of the paradigm shift: Global collective action

The persistence of country-income and stability categories, despite well-documented evidence of their inadequacy, is not primarily an intellectual failure - it is a political economy problem. These categories persist because they serve specific interests: bilateral donors rely on them to manage overseas development assistance flows; multilateral institutions with country-based lending portfolios depend on them to structure conditionality and repayment obligations; and governments assert sovereignty over domestic equity questions partly through income-category claims. These actors have strong incentives to maintain the current classification architecture, which is why the intellectual case against country categories is insufficient to displace them. Naming this is a prerequisite for effective advocacy: a framework that treats the problem as one of measurement will propose measurement solutions, while one that treats it as a political economy problem will propose coalition-building toward key reforms.

This foundation of collective action is founded in the historical record of social movements in health, reminding us that the shifts called for are neither unprecedented nor impossible. The Treatment Action Campaign in South Africa provides a powerful example of community-driven accountability forcing both a government and donors to reverse course on treatment access [35]. Together with the MSF-led Access to Medicines campaign and broader coalitions, their role in securing the 2001 Doha Declaration on TRIPS and Public Health demonstrate that transnational civil society can reshape structural conditions governing health equity [36,37]. These social movements make visible the organizational infrastructure underpinning the specific functional shifts proposed in our revised framework.

## Conclusion

A new global health architecture centered on equity requires, at the core, redistribution of knowledge, resources, and decision-making power - but this redistribution confronts the fundamental tension that states will not voluntarily cede authority to equity-oriented supranational frameworks. This tension is not fully resolvable within the current political order. The distinction is between “thin” and “thick” multilateralism, moving from a shallow focus on coordination to a deeper and real engagement with distributive justice.. The global agenda advanced in this Essay requires the latter, which is precisely why civil society mobilization and community-level accountability are not supplementary to the framework but constitutive of it. It is this local to global collective action that will tie together the shared struggles of marginalized communities across the world, regardless of whether they reside in low, middle, or high-income countries – including in rural Alabama, northern Australia, and the densely populated neighborhoods of London. A global health system that does not begin from a commitment to health equity - and the shifts in power it requires - risks, at best, perpetuating the inequities it claims to address, and at worst, actively deepening them.
